# Readability Analysis of Patient Education Material on Rotator Cuff Injuries From the Top 25 Ranking Orthopaedic Institutions

**DOI:** 10.5435/JAAOSGlobal-D-24-00085

**Published:** 2024-05-09

**Authors:** Michael Miskiewicz, Salvatore Capotosto, Kenny Ling, Frederick Hance, Edward Wang

**Affiliations:** From the Renaissance School of Medicine, Stony Brook University, Stony Brook, NY (Mr. Miskiewicz and Mr. Capotosto), and the Department of Orthopaedics, Stony Brook University Hospital, Stony Brook, NY (Dr. Ling, Dr. Hance, and Dr. Wang).

## Abstract

**Introduction:**

Rotator cuff injuries (RCIs) are incredibly common in the US adult population. Forty-three percent of adults have basic or below-basic literacy levels; nonetheless, patient educational materials (PEMs) are frequently composed at levels exceeding these reading capabilities. This study investigates the readability of PEMs on RCIs published by leading US orthopaedic institutions.

**Methods:**

The top 25 orthopaedic institutions on the 2022 U.S. News & World Report Best Hospitals Specialty Ranking were selected. Readability scores of PEMs related to RCI were calculated using the www.readabilityformulas.com website.

**Results:**

Among the 25 analyzed PEM texts, all exceeded the sixth-grade reading level. Only four of 168 scores (2.4%) were below the eighth-grade level.

**Discussion:**

This study indicates that PEMs on rotator cuff injuries from top orthopedic institutions are too complex for many Americans, with readability levels ranging from 8.5 to 16th grade, well above the CDC-recommended eighth-grade level. The research highlights a widespread issue with high reading levels across healthcare information and underscores the need for healthcare providers to adopt patient-centered communication strategies to improve comprehension and accessibility.

**Conclusion:**

PEMs on rotator cuff injuries from leading orthopedic institutions often have a reading level beyond that of many Americans, exceeding guidelines from the NIH and CDC that recommend PEMs be written at an eighth-grade reading level. To increase accessibility, enhance healthcare literacy, and improve patient outcomes, institutions should simplify these materials to meet recommended readability standards.

Patients are increasingly turning to online resources and patient educational materials (PEMs) to gain insights into common medical conditions and treatments. While convenient access to accurate information online can positively affect the patient-doctor relationship, its effectiveness relies on the patient's ability to read, interpret, and comprehend the presented information. Patient literacy poses a notable challenge to healthcare providers in effectively communicating through written PEMs.

Reports from the National Center for Education Statistics indicate that on average, Americans read at an eighth-grade reading level.^1^ Both the National Institutes of Health (NIH) and the Centers for Disease Control and Prevention (CDC) recommend that PEMs be written at a sixth- and eighth-grade reading level, respectively.^2,3^ Prior research has consistently demonstrated a correlation between poor health literacy and adverse clinical outcomes^4,5,6,7,8,9^; therefore, it is imperative for healthcare providers to communicate effectively with their target audience.

Rotator cuff injuries (RCIs) are one of the most common causes of shoulder pain and disability of the upper extremity.^9^ Older age, smoking, participation in sports, and occupations requiring overhead activity are among the many well-established risk factors of RCI.^9,10^ The management of RCI often consists of a multifaceted approach that includes anti-inflammatory medication, physical therapy, and, in some cases, surgery.^11^ Proactive management of RCI is critically important to prevent progressive worsening of the injury.^12^ It is important for surgeons to clearly communicate this with patients. Given the substantial research indicating the discrepancy between online PEM and national reading grade averages,^13,14,15,16,17,18^ we hypothesize that online PEMs related to RCI are written at a reading grade level that exceeds that of the NIH and CDC recommendations. Therefore, this study aims to evaluate the readability level of PEMs for RCI published by the leading national orthopaedic institutions and determine whether these PEMs consider the specific needs of this target audience.

## Methods

The top 25 orthopaedic hospitals in the country were identified based on the 2022 U.S. News & World Report Best Hospitals Specialty Ranking. Pertinent PEMs related to RCIs were gathered from the websites of each institution. Institutions lacking relevant PEMs were excluded from the study. The PEMs were then converted into a text-only format, and their readability scores were calculated using various tests available on the http://www.readabilityformulas.com website.

The readability tests, listed in Table 1, were used. These tests included the Flesch-Kincaid Reading Ease Score, Gunning Fog, Flesch-Kincaid Grade Level, Coleman-Liau Index, Simple Measure of Gobbledygook (SMOG) Index, Automated Readability Index, and Linsear Write Formula. The results of each test were used to calculate the mean and standard deviation for each institution. All output scores were reported as a corresponding grade-appropriate reading level, except for the Flesch-Kincaid Reading Ease Score. This provides values from 0 to 100, in which a higher number indicates an easier reading level. The readability categories for the Flesch-Kincaid scores are defined as follows: Scores from 90 to 100 signify texts that are very easy to understand, 80 to 89 denote easy reading, 70 to 79 are considered fairly easy, 60 to 69 represent standard difficulty, 50 to 59 are somewhat challenging, 30 to 49 categorize texts as difficult, and scores between 0 and 29 are labeled as confusing.

**Table 1:** Description of Readability Tools and Their Corresponding Formulas

Readability Tool	Formula
Flesch-Kincaid Reading Ease Score	Readability ease = 206.835 − (1.015 × average sentence length) − (84.6 × average number of syllables per word)
Gunning Fog	Grade level = 0.4 (average sentence length/percentage of hard words)
Flesch-Kincaid Grade Level	Grade level = (0.39 × average sentence length) + (11.8*average # syllable per word) − 15.59
Coleman-Liau Index	Grade level = 0.0855 (average # of letters per 100 words) − 0.296(average # of sentences per 100 words) − 15.8
SMOG Index	Grade level = 3 * square root of polysyllable count
Automated Readability Index	Grade level = 4.71 (characters/words) + 0.5(words/sentences) − 21.43
Linsear Write Formula	*n* = [(2 syllables words * 1) + (3 or more syllable words * 3)]/number of sentences If *n* < 20, grade level = *n*/20 If *n* > 20, grade level = *n* − 2/20

To gauge collinearity among the readability tests, variance inflation factors (VIFs) were computed. A VIF equal to or greater than 10 indicated substantial collinearity between tests and readability scores.^19^ Finally, Spearman regression modeling was used to explore the correlation between Flesch-Kincaid Reading Ease Scores and the ranking of institutions.

## Results

**Figure 1:**
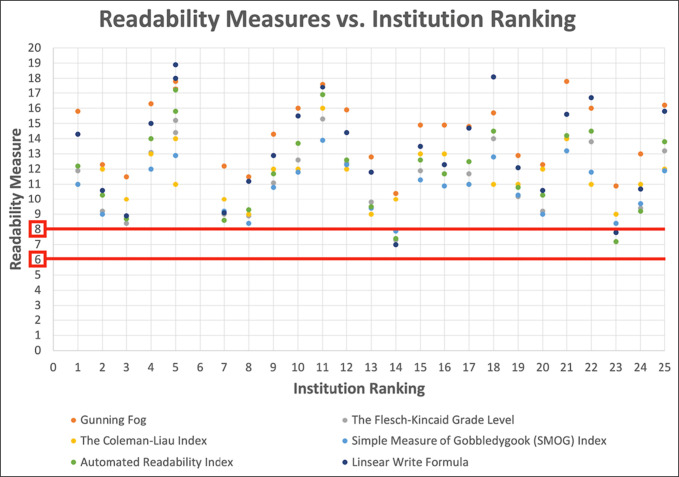
Graph showing Flesch-Kincaid grade readability scores on patient education materials on lateral epicondylitis from some of the top orthopaedic institutions in relation to the average American reading grade level. *Red lines denote the recommended patient educational material reading grade levels by the National Institutes of Health (sixth grade) and the Centers for Disease Control and Prevention (eighth grade).

All 25 orthopaedic institutions included in the study provided PEMs related to RCIs for analysis. The average readability score on the Flesch-Kincaid Reading Ease Scale was 48.4 ± 10.8 out of 100. Other scores were as follows: Gunning Fog, 14.4 ± 2.3; FK Grade Level, 11.4 ± 2.4; Coleman Liau Index, 11.6 ± 1.7; SMOG Index, 10.8 ± 1.7; Automated Readability Index, 12.0 ± 2.8; and Linsear Write Formula, 13.3 ± 3.3. A comprehensive summary of individual readability scores and institution rankings is provided in Table 2. Notably, only 2.4% of the PEMs from these institutions were composed at or below the eighth-grade reading level, in accordance with CDC recommendations (Figure 1). None of the PEMs met the recommended sixth-grade reading level, as advised by the NIH.

**Table 2:** Individual and Mean Grade Levels and Readability Ease for the Top 25 Orthopaedic Institutions

Institution Ranking	Flesch Reading Ease Score*	Gunning Fog	Flesch-Kincaid Grade Level	Coleman-Liau Index	SMOG Index	Automated Readability Index	Linsear Write Formula	Average (SD)
1	46.8	15.8	11.9	11.0	11.0	12.2	14.3	12.7 (1.8)
2	58.5	12.3	9.2	12.0	9.0	10.3	10.6	10.6 (1.3)
3	62.5	11.5	8.4	10.0	8.7	8.7	8.9	9.4 (1.1)
4	39.2	16.3	13.1	13.0	12.0	14.0	15.0	13.9 (1.4)
5	41.1	17.3	14.4	11.0	12.9	15.8	18.9	15.1 (2.6)
	32.2	17.8	15.2	14.0	12.9	17.2	18.0	15.9 (1.9)
7	57.7	12.2	9.0	10.0	9.2	8.6	9.1	9.7 (1.2)
8	64.0	11.5	8.9	9.0	8.4	9.3	11.2	9.7 (1.2)
9	49.2	14.3	11.1	12.0	10.8	11.7	12.9	12.1 (1.2)
10	45.2	16.0	12.6	12.0	11.8	13.7	15.5	13.6 (1.6)
11	26.6	17.6	15.3	16.0	13.9	16.9	17.4	16.2 (1.3)
12	41.0	15.9	12.4	12.0	12.3	12.6	14.4	13.3 (1.4)
13	57.4	12.8	9.8	9.0	9.4	9.5	11.8	10.4 (1.4)
14	64.4	10.4	7.3	10.0	7.9	7.4	7.0	8.3 (1.4)
15	44.2	14.9	11.9	13.0	11.3	12.6	13.5	12.9 (1.2)
16	42.3	14.9	11.7	13.0	10.9	11.7	12.3	12.4 (1.3)
17	50.9	14.8	11.7	11.0	11.0	12.5	14.7	12.6 (1.6)
18	41.6	15.7	14.0	11.0	12.8	14.5	18.1	14.4 (2.2)
19	53.9	12.9	10.2	11.0	10.3	10.8	12.1	11.2 (1.0)
20	58.5	12.3	9.2	12.0	9.0	10.3	10.6	10.6 (1.3)
21	31.7	17.8	14.0	14.0	13.2	14.2	15.6	14.8 (1.5)
	40.7	16.0	13.8	11.0	11.8	14.5	16.7	14.0 (2.1)
23	63.1	10.9	7.8	9.0	8.4	7.2	7.8	8.5 (1.2)
24	55.6	13.0	9.4	11.0	9.7	9.2	10.7	10.5 (1.3)
	41.8	16.2	13.2	12.0	11.9	13.8	15.8	13.8 (1.7)
Average (SD)	48.4 (10.8)	14.4 (2.3)	11.4 (2.4)	11.6 (1.7)	10.8 (1.7)	12.0 (2.8)	13.3 (3.3)	

SMOG = Simple Measure of Gobbledygook

* Flesch Reading Ease Score is out of 100. All remaining scores are grade-levels.

Scores above the 12th grade reading level indicate a college or graduate/professional level of readability.

Our analysis of collinearity indicated a substantial level of collinearity for each test in comparison with the Flesch-Kincaid Reading Ease Score. The VIFs for individual tests were consistently above 10, with the following values: Gunning Fog = 31.0, FK Grade Level = 144.7, Coleman Liau Index = 10.4, SMOG Index = 43.0, Automated Readability Index = 106.1, and Linsear Write Formula = 66.1. In addition, Spearman regression analysis revealed no significant correlation between institution ranking and the Flesch-Kincaid Reading Ease Score (ρ = 0.003; *P* = 0.99).

## Discussion

This study suggests that PEMs on RCIs published by the top 25 nationally ranked orthopaedic institutions are written between a 8.5th and 16th grade reading level. Additional findings of this study demonstrated a high degree of collinearity between individual readability scores. No notable correlation was found between institutional ranking and the Flesch-Kincaid Reading Ease Score.

Personal health literacy has been defined by the CDC as the degree to which an individual is capable of finding, understanding, and using information and services to guide health-related decision making.^23^ Past research has demonstrated a strong relationship between poor health literacy and poor health.^4,5,6,7,8^ Additional studies have estimated the national cost burden of limited health literacy to be between $106 and $236 billion annually.^24^ For these reasons, it is imperative for healthcare providers to adopt patient-centered approaches to communication that account for variations in health literacy. Tailored communication is an effective approach that has shown favorable outcomes in enhancing communication methods to suit a specific target patient group appropriately.^20,21,22,23,24,25,26^ Regardless, it is ultimately up to individual members of a healthcare organization to audit their own institution's communication modalities.

Our results demonstrate that none of the PEMs on RCIs from the top 25 ranking orthopaedic institutions in the nation had calculated readability scores that fall within the CDC-recommended guidelines. This finding is congruent with past research that has analyzed PEMs related to both orthopaedic and nonorthopaedic fields.^13,14,15,16,17,18^ A recent study by Para et al.^27^ used a similar methodology to evaluate the readability of online PEM related to orthopaedic oncologic pathologies. Their results demonstrated a notable discrepancy between the average readability scores of the orthopaedic oncologic PEMs analyzed and the national reading grade average. This pattern has been further characterized in other articles that broadened the scope of their search to include PEMs from additional sources, including orthopaedic implant manufacturing companies,^28^ the American Academy of Orthopaedic Surgeons website,^29,30^ and the Pediatric Orthopaedic Society of North America website.^30^ The notable divergence between the readability levels of PEMs analyzed throughout the literature and national reading averages highlights the extensive nature of this problem. As such, it calls for comprehensive initiatives aimed at improving the clarity and comprehension of PEMs to better serve diverse patient populations.

Our study has some limitations. First, the assessment of readability scores serves as just one aspect in characterizing the complexity of PEMs. Factors like the incorporation of audiovisual multimedia or the overall layout and design of websites could not be comprehensively examined in this study. Moreover, our analysis specifically concentrated on PEMs from the top 25 orthopaedic institutions, based on the 2022 U.S. News & World Report Best Hospitals Specialty Ranking. It is important to acknowledge that these PEMs and their associated readability levels might not fully represent those provided by other orthopaedic institutions across the country. Finally, our study only analyzed PEMs written in English. Future research investigating readability of similar articles in varying languages may shed insight into the global variability in comprehension and accessibility of PEMs, potentially identifying linguistic and cultural barriers to effective healthcare communication. We encourage administrative faculty nationwide to critically assess the PEMs within their own institutions and make necessary modifications as needed.

## Conclusion

The findings of this study highlight that PEMs concerning RCIs from the top-ranked orthopaedic institutions can exceed a reading level of a large number of Americans. In alignment with guidelines from the NIH and CDC, orthopaedic institutions should aim to create PEMs that are accessible at or below an eighth-grade reading level. Enhancing the readability of PEMs can broaden outreach, enhance healthcare literacy, and ultimately contribute to improved patient outcomes.
